# Change is in the air: key questions on the ‘Treatable Traits’ model for chronic airway diseases in primary care

**DOI:** 10.1038/s41533-024-00381-y

**Published:** 2024-07-18

**Authors:** Alvar Agusti, Peter G. Gibson, Liam G. Heaney, Mike Thomas

**Affiliations:** 1 Respiratory Institute, Clínic Barcelona, Barcelona, Spain; 2 Catedra Salud Respiratoria, University of Barcelona, Barcelona, Spain; 3 FCRB-IDIBAPS, Barcelona, Spain; 4 CIBER Enfermedades Respiratorias, Barcelona, Spain; 5 Asthma and Breathing Research Centre and Hunter Medical Research Institute, Faculty of Health and Medicine, The University of Newcastle, Callaghan, NSW Australia; 6 Wellcome-Wolfson Institute of Experimental Medicine, School of Medicine, Dentistry and Biomedical Sciences, Queen’s University Belfast, Belfast, UK; 7 Primary Care Research Centre, School of Primary Care, Population Sciences and Medical Education (PPM), Faculty of Medicine, University of Southampton, Southampton, UK

**Keywords:** Respiratory tract diseases, Health care

## Abstract

Despite great advancements in the treatment of chronic airway diseases, improvements in morbidity and mortality have stalled in recent years. Asthma and chronic obstructive pulmonary disease are complex and heterogeneous diseases that require tailored management based on individual patient characteristics and needs. The Treatable Traits (TTs) approach aims to personalise and improve patient care through the identification and targeting of clinically relevant and modifiable pulmonary, extra-pulmonary and behavioural traits. In this article, we outline the rationale for TTs-based management and provide practical guidance for its application in primary care. To aid implementation, seven potential ‘prime’ traits are proposed: airflow obstruction, eosinophilic inflammation, adherence, inhaler technique, smoking, low body mass index/obesity and anxiety and depression—selected for their prevalence, recognisability and feasibility of use. Some of the key questions among healthcare professionals, that may be roadblocks to widespread application of a TTs model of care, are also addressed.

## Introduction

Asthma and chronic obstructive pulmonary disease (COPD) are two prevalent, complex and heterogeneous airway diseases with clinical features that may overlap^1,2^. In recent years, a management paradigm based on the identification of so-called ‘treatable traits’ (TTs) has been proposed to personalise and improve patient care. The TTs strategy targets clinically relevant, measurable and modifiable characteristics (traits) in each patient in the pulmonary, extra-pulmonary and behavioural and psychosocial domains^3^. The TTs approach is agnostic to traditional disease labels and applicable to all patients with chronic airway diseases^4^. By recognising the complexity and variability in clinical and biological characteristics among individual patients with chronic airway diseases, treatment is more rational, precise and effective.

Although the concepts underpinning the TTs approach are now well established, some areas of uncertainty remain among healthcare professionals (HCPs), particularly in the primary care setting. Below, we discuss *(1)* the rationale for introducing a TTs approach in clinical practice; and, *(2)* the hurdles and challenges of its implementation.

## Rationale for implementing a Treatable Traits approach in clinical practice

### Conceptual advantages

Asthma and COPD guidelines have traditionally advocated a stepwise approach to adjusting treatment based on symptom control and reducing the risk of exacerbations^1,2^. This has been instrumental in improving outcomes for patients, as shown by reductions in hospital admission and mortality in the 1990s to early 2000s^5^. However, progress has stalled with no overall improvements to asthma and COPD mortality observed between 2001 and 2017^5,6^. This slowed progress may be attributed to the fact that guidelines are largely informed by evidence derived from grouped mean data from randomised controlled trials (RCTs). This may result in treatment algorithms biased towards the greatest efficacy for the majority, but not necessarily the most appropriate treatment for each individual patient^5^. Further, the stepwise approach assumes that *(1)* symptoms are driven by the same mechanisms in individual patients, and *(2)* symptom control is achieved by following the ‘one-size-fits-all’ treatment algorithm, although this is not always the case^7^. In fact, in many patients, elimination of symptoms and restoration of normal lung function cannot be achieved^8,9^ and pursuing this unattainable goal by progressively increasing treatment may lead to patient and clinician frustration, as well as adverse effects from high-dose treatment. This is particularly pertinent for inhaled corticosteroids (ICS), where 80–90% of the maximal therapeutic effect may be achieved at low doses in most patients^10^. Escalation to higher doses of ICS should be preceded by both confirmation of adherence to treatment and the presence of the relevant biological processes targeted by corticosteroids (elevated blood eosinophils or fractional exhaled nitric oxide [FeNO]). In addition, current asthma guidelines advocate stepping down treatment when asthma symptoms are controlled. While stepping down treatment may be appropriate in certain circumstances, a universal step-down model risks the false assumption that treatment targets and disease characteristics are homogenous in all patients^7^. It also assumes that a short-term increase in treatment can, in some way, change the underlying disease pathophysiology to reduce future requirement for treatment. By contrast, in COPD, the Global Initiative for Chronic Lung Disease (GOLD) recommends assessing two key TTs (dyspnoea and exacerbations) to manage patients during follow-up and rarely recommends stepping down treatment, with the exception of patients on ICS who suffer from, or are at risk of, pneumonia, in whom stopping ICS is an option for consideration^2^.

The heterogeneity, complexity and multi-morbidity in each patient needs to be considered on an individual basis^11^. The TTs approach offers a practical method of designing personalised treatment strategies that account for patient heterogeneity. This is not a revolutionary concept, nor is it inherently complex; rather, it reinforces many existing elements of good clinical practice—identifying and treating clinical features presented in an individual patient. Analogous approaches have been implemented in other areas with great success, such as cardiovascular disease. During the 1990s, heart attack risk factors, such as blood pressure, cholesterol and smoking, were stratified into a theragnostic tool for predicting and preventing future events and this vastly improved clinical outcomes^12^.

### Real-life examples of the Treatable Traits approach in clinical practice

#### Use of oral corticosteroids in asthma and COPD

Rescue oral corticosteroids (OCS) play an important role in the acute management of exacerbations of asthma and COPD^1,13,14^. However, it is well established that long-term use of OCS is associated with side effects^1,15–18^. It was found that patients with severe asthma who received frequent prednisolone (≥4 prescriptions per year) had an increased risk of many conditions associated with OCS exposure, with the highest risk observed with maintenance daily prednisolone^19^. Additional studies have shown that OCS-induced morbidity emerged at low levels of exposure and validate that episodic rescue prednisolone is not a benign intervention^15,16^. However, OCS prescription remains widespread and use in the treatment of poorly controlled mild–moderate disease is particularly concerning^18^. Doses of OCS can be minimised through the identification of biomarkers and TTs within a structured management approach, to optimise treatment and reduce the risk of exacerbations^18^. Following this approach, there is an initial assessment of disease severity, after which comorbidities and risk factors contributing to difficult-to-treat asthma are identified and treated. If this proves ineffective, referral to a specialist is advised before consideration of prescribing OCS at the minimum dose^18^.

#### Measuring eosinophilic airway inflammation

Type 2 (T2) airway inflammation has been identified as a TT in some patients with asthma or COPD, and can be assessed by measurement of blood eosinophil counts and/or FeNO (if available) (Table 1). Eosinophilic airway inflammation is associated with an increased exacerbation risk and more severe disease in patients with chronic airway diseases^1,2^. In asthma, eosinophilic airway inflammation is mediated by allergic sensitisation and T2 lymphocytes, although eosinophilic inflammation can occur independently of an allergic response. The eosinophilic phenotype is common, although not universal, with ~50–70% of patients having T2-high asthma^20–22^ and this estimate is higher in a severe asthma population (>80%)^23^. In contrast, airway inflammation in COPD is typically associated with a neutrophilic response (Type 1) and 20–40% of patients with COPD also present with an eosinophilic phenotype^22,24^. Therefore, the presence of T2 inflammation requires assessment as part of phenotype and risk determination^22^.

**Table 1:** Prime treatable traits and their treatment outcomes.

Treatable trait	How to identify the trait in primary care	Impact on disease burden	Treatment	Evidence	
				Asthma	COPD
Airflow obstruction/limitation	Spirometry test to confirm FEV_1_/FVC is below the lower limit of normal (usually >0.75–0.80 in adults, >90 in children)^1,2^	Airflow obstruction is associated with increased lung function decline, and limited exercise capacity^1,39,40^	Airflow obstruction can be treated with bronchodilators, such as LAMA and/or LABA, in COPD, and LAMA and/or LABA in combination with ICS, in asthma^1,2^	Treatment with ICS-formoterol has demonstrated a reduced risk of exacerbations compared with ICS, while still maintaining symptom and FEV_1_ control, and is recommended for the initial treatment of mild asthma^1^	LABA and LAMA can significantly improve FEV_1_ and lung volumes, as well as dyspnoea and exacerbation rate^2,41^
Eosinophilic inflammation	Measure eosinophilia in blood or FeNO in breath^1,57^	Eosinophilic inflammation is directly linked to an increased risk of exacerbations and uncontrolled asthma^36^	ICS can be used to treat eosinophilic inflammation, and blood eosinophil levels are effective in predicting response to ICS in COPD and asthma, as well as biologic therapy in asthma^4,34,35,55^	Treatment of mild asthma with ICS-formoterol reliever therapy has been shown to reduce the rate of exacerbations by 60% when compared with SABAs^54^	Pooled analysis of 7,495 patients across 7 studies demonstrated that treatment with ICS-LABA reduced the frequency of exacerbations compared with placebo in patients with mild to very severe COPD (RR 0.73; 95% CI, 0.69–0.78)^58^
Adherence	Open discussion between HCPs and patients, checking prescription refill rate, and use of ‘smart’ chipped inhaler technology^1,4^	Increased risk of uncontrolled asthma and COPD^1,2^	Various strategies have been established to improve adherence: shared decision-making for medication dosage, inhaler reminders and home visits^1^	A systematic literature review found that greater adherence is associated with a higher FEV_1_, a reduced risk of hospitalisation, a lower percentage of sputum eosinophils and reduced OCS dependency^47^	Improved adherence is associated with a reduced risk of admission to hospital and mortality due to a COPD exacerbation^59^
Inhaler technique	Observation of inhaler technique and use of training devices^4^	Worsening of symptoms and an increased risk of exacerbations^1^	HCP-led training and development of smart inhalers to provide immediate feedback to patients^49,60^	Patient errors when using inhalers could cause suboptimal drug delivery^49^ and, in turn, result in higher risks of hospitalisation (OR 1.47), ER visits (OR 1.62) and OCS use (OR 1.54)^61^. After training, the percentage of asthma patients with optimal inhaler technique has been shown to rise from 24% to 79% (*p* < 0.001)^60^. Furthermore, correct inhaler technique has the combined benefit of improving asthma control and treatment response to ICS^1^	
Smoking	Open discussion between HCPs and patients. Exposure can be measured by cotinine or exhaled concentration of carbon monoxide^4^	Smoking is the main risk factor for COPD^2^. It is also associated with an increased rate of lung function decline and reduced responsiveness to ICS and OCS in asthma^1,29^, as well as worse outcomes in both conditions^1,2^	Education and smoking cessation support, a quit plan with social support and clinician counselling^1,2^	A study found patients to have significantly increased lung function 6 weeks after smoking cessation^50^	Lung function decline is slowed in patients following smoking cessation^51^. Smoking cessation by the age of 40 years significantly reduces the risk of mortality in patients with COPD^62^
Low BMI/obesity	Documentation of BMI for all patients, as well as assessment of diet and exercise^1^	Obesity can contribute to worse symptom control in patients with asthma^1^. A low BMI in patients with COPD is associated with worse outcomes^2^	Exercise and a healthy diet^1^	After just a 5–10% body weight loss among overweight and obese patients, evident improvement to asthma control has been reported^63^	Malnutrition and weight loss in patients with COPD was found to be significantly associated with disease severity (*p* = 0.039). An extra one meal a day was observed to improve QoL, with a 3.61 decrease in SGRQ score^64^
Anxiety and depression	Questionnaires and an assessment with a psychiatrist or liaison psychiatrist^1,4^	Anxiety and depression are associated with worse symptom control, poor adherence and increased exacerbations in patients with asthma^1^. Anxiety and depression contribute to fatigue, poorer exercise tolerance and higher frequency of acute exacerbations in patients with COPD^65^	CBT, pharmacotherapy, mind-body interventions and other psychotherapies are among management options for anxiety and depression^1,2^	Patients with asthma and comorbid anxiety who received 4–6 1-h weekly sessions of CBT had a statistically significant reduction in asthma-specific fear when compared with routine treatment; this was maintained at a 6-month follow-up. In addition, significant improvements in asthma-specific QoL and depression were observed in CBT groups, with a 5% reduction in the number of patients with possible clinical depression at the end of treatment^52^	Mind-body interventions have been shown to improve lung function and exercise capacity in patients with psychological comorbidities^2^

*BMI* body mass index, *CBT* cognitive behavioural therapy, *CI* confidence interval, *COPD* chronic obstructive pulmonary disease, *ER* emergency room, *FEV*_*1*_ forced expiratory volume in 1 s, *FVC* forced vital capacity, *HCP* healthcare professional, *ICS* inhaled corticosteroids, *LABA* long-acting β_2_-agonist, *LAMA* long-acting muscarinic antagonist, *OCS* oral corticosteroids, *OR* odds ratio, *QoL* quality of life, *SABA* short-acting β_2_-agonist, *RR* rate ratio, *SGRQ* St George’s Respiratory Questionnaire.

Sputum eosinophil count is an effective indicator of eosinophilic airway inflammation, but it is not broadly used in clinical practice due to duration of the analysis and required expertise and facilities^1,25,26^. Alternatively, blood eosinophil count is an effective and more practical marker of eosinophilic inflammation^26,27^. Measuring blood eosinophils is a simple, inexpensive and quick method that is frequently performed in primary care^27^. FeNO testing can also be used to assess the presence of eosinophilic airway inflammation, although its accessibility is generally confined to the specialist domain^26,28^. Furthermore, controversy persists within treatment guidelines as to the role of FeNO in the management of asthma in primary care. The British Thoracic Society and European Respiratory Society guidelines have recently advised that FeNO may have a role in asthma diagnosis and clinical decision-making^29,30^. A recently updated systematic review has demonstrated that FeNO-guided treatment may reduce exacerbation frequency in patients with asthma, though the clinical relevance of these effects could not be determined^31^. FeNO suppression testing with monitored ICS treatment is useful in distinguishing patients who require escalation to biologic therapy from those in which improved adherence to ICS is likely to achieve disease control^32–34^, and has been shown to be cost effective^35^. It is not yet clear whether routine FeNO testing in all patients with asthma is clinically effective or cost effective, this may be elucidated by a large UK programme currently exploring its suitability in primary care^36^.

By contrast, there is a growing volume of evidence that indicates blood eosinophils are a predictive biomarker for responsiveness to ICS in patients with asthma and COPD. Studies have demonstrated a continuous beneficial effect in reduction of exacerbation rate from ICS treatment in correlation with increasing blood eosinophil count in patients with COPD^37^. This evidence has led to inclusion of eosinophil measurement in international guidelines as a clinical decision-making factor. The GOLD strategy report recommend using a blood eosinophil count of ≥300 cells/μL as an indication for escalation of treatment from a long-acting muscarinic antagonist (LAMA) + long-acting β_2_-agonist (LABA) combination to triple therapy treatment (ICS + LAMA + LABA)^2^. Similar utility of the biomarker is found in asthma; greater treatment benefits are observed in patients with eosinophilic asthma treated with ICS versus patients with lower blood eosinophil count, with regard to exacerbation rate, lung function and health-related quality of life^38,39^. However, utilisation of blood eosinophil count as a predictive marker for targeting ICS use in patients with asthma is not yet recommended by the Global Initiative for Asthma (GINA)^1^. In cases of severe asthma which remain uncontrolled despite optimised therapy, patients should be referred to a specialist for further investigation. Severe eosinophilic asthma can be effectively treated in the specialist domain with add-on T2-targeting biologic therapy, to which blood eosinophil count is a well-documented and reliable predictor of responsiveness^1^. GINA recommendations advocate a blood eosinophil level threshold of ≥150 cells/μL for consideration of add-on T2-targeting biologic therapy in patients with exacerbations or poor symptoms control^1^. In summary, eosinophilic inflammation is an important TT to address; its widespread measurement in primary care may inform rational treatment decisions and facilitate timely, appropriate referrals for specialist assessment^40,41^.

### Patient engagement, education and empowerment

Some common frustrations among patients with asthma and COPD regarding the current treatment paradigm include a feeling that their own beliefs and concerns are not recognised and a perception that the treatment is neither patient-centric nor tailored to their personal needs and goals^42^. Together, these frustrations may erode the patient–clinician partnership, diminish engagement and contribute to non-adherence to treatment^42^. The TTs approach, however, empowers prescribers to avoid inappropriate treatment when specific traits have been ruled out. It provides patients with a demonstrable common-sense fit between their understanding of the problem (drivers of disease) and the proposed solution (treatment plan)^4,12^. In the same way that patients with cardiovascular disease may know their blood pressure or cholesterol numbers and patients with type 2 diabetes may know their haemoglobin A1c, the TTs model of care, helps patients with asthma or COPD understand their disease and make better-informed decisions about their medication use. This targeted approach enables collaborative decision-making with patients, with rationally agreed treatment goals^12^. In summary, by avoiding patient frustration due to misaligned treatment expectations, this could in turn promote good self-management and adherence. Finally, there is a perception that the TTs approach is complicated and may be overwhelming for patients^12^. By contrast, studies have shown that patients desire increased objective assessment, more individualised approaches and involvement in shared decision-making^12,42^.

## Practical implementation questions

There remains some uncertainty and hesitancy around the feasibility of implementing a TTs approach within current healthcare systems^12^; some common questions are discussed below.

### Are there a few ‘prime’ treatable traits that should be considered for the initial management of patients with chronic obstructive airways diseases?

There are many potential traits to consider in patients with airways diseases^4^. Simplification of the approach, to ease implementation in primary care, could have a substantial impact on early care and timely referrals. Therefore, we propose seven potential ‘prime traits’ (Table 1), selected for their prevalence, recognisability and feasibility of implementation in primary care. We also acknowledge that, in clinical practice, other TTs may deserve attention and treatment if identified in individual patients, such as rhinitis, gastroesophageal reflux, poor indoor and outdoor air quality (e.g. damp walls and/or presence of moulds), cardiovascular diseases and diabetes^1,12,43–46^.

1. *Airflow limitation* measurement is essential for the diagnosis and assessment of severity of chronic airway diseases^1,47,48^. With resources available to support training and equipment, quality-assured spirometry tests can be accurately performed in primary care^1,2,4,49^.
2. *Eosinophilic, or T2, inflammation* which, as previously discussed, may be measured by blood eosinophil count, is highly associated with exacerbation risk and is predictive of response to ICS and biologic therapy in COPD and asthma^1,2,39,40,50^. In addition, FeNO is a simple point-of-care breath test that could potentially be measured in primary care and is prognostic for exacerbation risk in asthma, particularly when combined with blood eosinophil count^31,41,51^. FeNO is also predictive of response to ICS and a useful tool for identifying poor ICS adherence in uncontrolled disease^28,52,53^. Although the utility of FeNO testing in primary care remains under investigation, if available, it is potentially valuable in implementing a TTs strategy for asthma^36,54^.
3. *Treatment adherence* is an essential driver of improved outcomes for both asthma and COPD^55,56^ and can be evaluated in a primary care setting through open discussions and review of the prescription refill rate^1,4^.
4. *Incorrect inhaler technique* is a common problem that reduces the effective delivery of drug(s) to the lung, with a detrimental impact on symptoms and exacerbation risk^1,57^. Outcomes may be improved through education and training on correct inhaler technique^1^.
5. *Smoking* should be assessed in each patient, with support and advice offered to encourage cessation^1,2,58,59^.
6. *Obesity* is a major contributor to poor asthma control; thus, *body mass index* (BMI) should be evaluated, and lifestyle and dietary interventions introduced where necessary^1^. In patients with COPD, low BMI is associated with worse outcomes and nutritional supplementation may be beneficial in those who are malnourished^2^.
7. Patients with chronic airway diseases are at increased risk of *anxiety and depression*, which are associated with reduced symptom control and an increased risk of exacerbations^1,2,60^. Mood disorder substantially impacts symptom burden, particularly in females^61^.

Importantly, many TTs are dynamic and their expression and relevance to the clinical situation may change—both with time and with effective treatment. Monitoring of traits is therefore needed, the frequency of which should be tailored to the individual patient depending on the cumulative effect of their particular traits. Furthermore, TTs do not always exist independently of one another; a change in one trait can, in some cases, effect change in another. For example, treating eosinophilic inflammation can reduce the frequency of exacerbations^39,62^. In fact, several factors, including exacerbations and dyspnoea, may be regarded as both traits and outcomes. This is important to consider when conducting routine observation of traits to enable effective management of clinically relevant changes over time.

### Implementation of a Treatable Traits approach can be done irrespective of the disease label

Disease labels are not required for identifying key traits or underlying drivers of disease. However, prescribing practices and healthcare pathways are generally reliant on the presence of a disease label. These labels certainly have value as the first step in diagnosis, but not the last, as we believe this should be augmented by a subsequent TTs assessment. For example, in the case of a patient presenting with the traditional disease label of ‘asthma’, who has a high level of blood eosinophilia, low forced expiratory volume in 1 second (FEV_1_) and an inconsistent prescription refill rate, under the TTs model a clinician could provide a more detailed diagnosis of ‘eosinophilic asthma with airflow obstruction and low adherence’. In this way, the diagnostic process is refined to provide a nuanced label for the individual patient, which is more meaningful for the patient and clinician alike. Another example relates to a patient with a diagnosis of asthma who is currently prescribed low doses of ICS without long-acting bronchodilators. In this case, the persistence of inadequate disease control plus a raised Th2 biomarker (e.g. FeNO) may indicate that increasing anti-inflammatory treatment, or addressing inadequate adherence/inhaler technique in the current ICS regime, could be effective management options. Finally, a high symptom report despite good lung function and normal biomarkers may indicate other factors, such as undiagnosed anxiety or depression, as more important targets than physiological or inflammatory factors for gaining control and improving wellbeing in a particular patient. These examples show that a TTs strategy would lead to more comprehensive treatment of the patient’s overall condition than if the clinician had focused solely on the traditional disease label. We believe an important evolution in respiratory care will be the interpretation of labels as the basis for further disease investigation, rather than a signpost towards a blanket one-size-fits-all approach to treatment.

### Is a controlled or real-world effectiveness trial needed to demonstrate the clinical benefit of the Treatable Traits approach?

A common question is around whether a RCT is needed to demonstrate the efficacy of the TTs model of care as a whole. It is notable that, historically, our treatment paradigms across airways diseases have not been based on any singular overarching study of the whole framework, but on individual controlled studies that demonstrate the efficacy of each component on managing an element of disease. Our guidelines have evolved iteratively as individual pieces of evidence became available. With this in mind, there are several reasons why a controlled efficacy trial for the TTs model of care may not be necessary. Firstly, the clinical impact of managing each individual trait is well supported by high-quality evidence, often from RCTs (see Table 1). For example, large studies have shown that eosinophilic inflammation in patients with asthma or COPD can be effectively treated with ICS and the level of inflammation is predictive of treatment response, with reductions in exacerbation rates increasing in proportion with blood eosinophil count^38,39,63^. Likewise, there is clear evidence that bronchodilators can be used to treat airflow obstruction; treatment with formoterol and salmeterol has demonstrated significant improvements in FEV_1_ in patients with COPD^49^. Secondly, an efficacy trial for the TTs approach may not be feasible logistically—to assess the efficacy of all prime traits simultaneously in a controlled setting, while comparing TTs to a standard model of care, in a sufficiently large patient population, would be prohibitively complex and time-consuming. Due to the inherent heterogeneity of airways diseases, controlling for multiple factors would also be an enormous challenge.

On the other hand, effectiveness trials have been performed in specific settings to investigate the relative benefits of the TTs approach compared with traditional stepwise treatment. McDonald et al. carried out a study to determine the effectiveness of a TTs approach for severe asthma in comparison with a stepwise approach to treatment. Eligible patients (*n* = 55) were randomised to receive either usual care or a targeted intervention based on a TTs approach, over a 16-week treatment period. The targeted intervention consisted of individualised biomarker-informed treatment and a multidisciplinary treatment plan guided by the identified TTs in each patient. The primary outcome was health-related quality of life, as assessed by the Asthma Quality of Life Questionnaire (AQLQ). The results of this study demonstrated a statistically and clinically significant improvement in health-related quality of life in the targeted intervention group, with a mean increase in AQLQ score of 0.86 from baseline (*p* < 0.001), compared with a mean value of −0.004 in the usual care group, which represented no significant change^64^.

A possible methodology to compare different types of individualised care could comprise a study in which a stacked approach—in which all seven prime traits are evaluated and treated in parallel—is compared with sequential assessment and treatment of traits to determine which leads to quicker disease control. Further studies could investigate prime traits versus a wider array of traits, and a specialist TTs service versus a solo practitioner aided by a practice nurse, as well as comparing different funding models. Findings of such studies could help to facilitate widespread acceptance and implementation of a TTs approach.

### When should treatable traits be assessed in clinical practice

We believe that a strategy based on TTs can and should be applied from the first visit and continued during follow-up. Of course, some TTs (e.g. adherence and inhaler technique) can only be applied during follow-up, after the patient has initiated therapy, but others can be assessed from the very first visit (e.g. symptoms, smoking, exacerbations and comorbidities).

### Cost of implementation

Finally, another persistent concern relates to potential costs associated with implementation of the TTs approach. We anticipate that, after an initial investment into clinical investigations, lifetime costs will likely be reduced due to potential improvement in exacerbation rate and clinical outcomes^12^, and from the improved cost-effectiveness of rationally targeted interventions rather than ‘trials of treatment’.

### Challenges in implementation in low-middle income countries

We recognise that there may be specific challenges for implementation of a TTs strategy in low-middle income countries (LMICs)^65^, including: *(1)* limited access to spirometry^66^. Hence, evaluation of airflow limitation is challenging; *(2)* although not exclusive to LMICs, clinicians in these settings may not have sufficient time with patients at consultations, which can negatively impact communication^67^. Hence, patient engagement, education and empowerment may be perceived as challenging; *(3)* access to FeNO testing is currently very limited^68^; *(4)* obtaining blood tests, such as eosinophil counts, may have financial implications for patients as, in many settings, healthcare is privatised and government resources are limited^66^. Further, the eosinophil count cut of 300 cells/μL may not be applicable to some LMICs^69^. Using this cutoff may lead to a higher proportion of patients being on ICS and may increase the risk of pneumonia in this population^70^. Despite these challenges, we believe that a basic TTs approach is feasible, even in such settings and that the improved targeting of treatment options is likely to prove clinically effective and cost effective in the wider perspective.

## Conclusions

The burden of disease for asthma and COPD remains high because of the complexity and heterogeneity of these diseases. It is time to advance towards the identification and targeting of TTs in order to treat the individual patient and look beyond the disease label they have been assigned.

### Reporting summary

Further information on research design is available in the Nature Research Reporting Summary linked to this article.

### Supplementary information

Reporting Summary
